# Examining the freezing process of an intermediate bulk containing an industrially relevant protein

**DOI:** 10.1016/j.enzmictec.2015.01.003

**Published:** 2015-04

**Authors:** Holger Reinsch, Oliver Spadiut, Johannes Heidingsfelder, Christoph Herwig

**Affiliations:** a Institut für Luft- und Kältetechnik gemeinnützige Gesellschaft mbH, Dresden, Germany; b Vienna University of Technology, Institute of Chemical Engineering, Research Area Biochemical Engineering, Vienna, Austria; c Laboratory for Mechanistic and Physiological Methods for Improved Bioprocesses, Institute of Chemical Engineering, Vienna University of Technology, Getreidemarkt 9/166, A-1060 Vienna, Austria

**Keywords:** Intermediate bulk, Pharmaceutical freezing, Product loss, pH shift, Physico-chemical changes, Biopharmaceutical processing

## Abstract

Numerous biopharmaceuticals are produced in recombinant microorganisms in the controlled environment of a bioreactor, a process known as Upstream Process. To minimize product loss due to physico-chemical and enzymatic degradation, the Upstream Process should be directly followed by product purification, known as Downstream Process. However, the Downstream Process can be technologically complex and time-consuming which is why Upstream and Downstream Process usually have to be decoupled temporally and spatially. Consequently, the product obtained after the Upstream Process, known as intermediate bulk, has to be stored. In those circumstances, a freezing procedure is often performed to prevent product loss. However, the freezing process itself is inseparably linked to physico-chemical changes of the intermediate bulk which may in turn damage the product.

The present study analysed the behaviour of a Tris-buffered intermediate bulk containing a biopharmaceutically relevant protein during a bottle freezing process. Major damaging mechanisms, like the spatiotemporal redistribution of ion concentrations and pH, and their influence on product stability were investigated. Summarizing, we show the complex events which happen in an intermediate bulk during freezing and explain the different causes for product loss.

## Highlights

• A Tris-buffered intermediate biopharmaceutical bulk was investigated during freezing.
• Media components and buffer salts experienced great concentration effects.
• pH showed local minima and maxima in the freezing good.
• Product loss was caused by a synergistic effect of local ion concentrations and pH values.

## Graphical abstract

The present study shows that freezing of a buffered intermediate bulk containing a biopharmaceutically relevant protein during a bottle freezing process describes a very complex process where product loss is caused by a synergistic effect of ion redistribution and pH changes. Only if the local redistribution of both, ion concentrations and pH values, are known, product loss may be calculated and minimized by optimizing the freezing process.

## 1 Introduction

Numerous industrially and medically relevant molecules are recombinantly produced in microorganisms. These bioprocesses usually comprise a production step in the controlled environment of a bioreactor, known as Upstream Process, and a subsequent product purification step, known as Downstream Process. Since the Downstream Process can be rather time-consuming involving several filtration and chromatography steps, it usually has to be temporally and spatially decoupled from the Upstream Process. In these cases, a freezing step of the intermediate product solution is necessary to ensure long term product stability during storage and transport [1]. This freezing step, however, affects the physico-chemical properties of the intermediate bulk potentially resulting in product loss. Despite this imminent risk, the freezing procedure is still a rather unexplored process step. Although studies on single aspects of freezing, like the effect of freezing and thawing rates, the use of different buffers and the addition of cryoprotectives, have been performed [2–6], mechanistic interactions of these aspects and resulting effects have not been investigated yet. However, without understanding and considering the complex interactions during a freezing process, a targeted product-specific optimization of the freezing procedure is not possible.

In the present study, we investigated the following scenario: a biopharmaceutically relevant protein, the enzyme horseradish peroxidase (HRP; EC 1.11.1.7; [7–10]), which is currently used in numerous medical applications [9–11], is extracellularly produced using recombinant yeast in a bioreactor. At the end of cultivation, the cell-free cultivation broth containing HRP is diafiltrated into a Tris/HCl buffer system, which describes a common buffer in industrial practice. At this stage the protein solution is called intermediate bulk. Since the Downstream Process for HRP is cumbersome, comprising several steps [12–15], it cannot be performed directly after diafiltration, which is why a freezing preservation step is required to prevent product loss. However, in addition to HRP and contaminating proteins, the intermediate bulk contains different components of the yeast cultivation broth, like salts and trace elements, which might experience concentration shifts during freezing and thus harm the product. The present study investigates the spatiotemporal redistribution of the dissolved intermediate bulk components and consequent pH-shifts during freezing and resulting effects on the product HRP in the 1 L scale using a standard lab freezer.

## 2 Materials and methods

### 2.1 Formulation of the intermediate bulk

The composition of the intermediate bulk was derived from a typical cultivation medium for *Pichia pastoris* [16], which is a suitable host for recombinant HRP production [17–21]. After separation of the biomass, the cell-free cultivation broth was three times diafiltrated with a 0.1 M Tris/HCl storage buffer. The Tris/HCl buffer system is suitable for HRP in terms of buffering capacity and pH range and is thus commonly used for processing this enzyme. Subsequently, a quantitative analysis of the bulk composition was performed. Table 1 shows the concentrations of all bulk components after diafiltration. This medium was reproduced to perform freezing experiments. Residual trace elements were not considered because their concentrations were too low to potentially damage the product. For freezing studies, we added a commercially available HRP preparation (SIGMA; type VI-A) at a concentration of 50 mg/l, which is a common concentration at the end of fed-batch production processes [19,20]. With respect to an acceptable degree of complexity, the freezing study was done without addition of stabilizers or cryoprotective agents.

### 2.2 Characterization of HRP in aqueous solution

#### 2.2.1 Enzymatic activity and protein content

The activity of HRP was determined photometrically using a CuBiAn XC enzymatic robot (Innovatis, Germany). Samples (10 μl) were added to 140 μl of 1 mM ABTS prepared in 50 mM KH_2_PO_4_ buffer (pH 6.5). The reaction mixture was incubated at 30 °C and started by the addition of 20 μl of 0.075% (v/v) H_2_O_2_. Changes of absorbance at 415 nm were measured and rates were calculated. Calibration was done using commercially available HRP as standard (0.02–1.0 U/ml). Protein concentrations for all tests in aqueous solution were determined by the Bradford assay [22] with bovine serum albumin (BSA) as standard.

#### 2.2.2 Stability at 30 °C

To exclude the possibility that already the incubation at 30 °C during enzyme activity assay might affect protein stability, HRP was dissolved in KH_2_PO_4_ buffer (50 mM, pH 6.5) to a concentration of 1 U/ml and incubated at 30 °C for up to 8 h. Samples were taken every 15 min and catalytic activity was determined. All samples were measured in triplicates.

#### 2.2.3 pH stability

To check pH stability, HRP was dissolved in different buffer systems to a final concentration of 1 U/ml, incubated at 30 °C for 30 min and remaining catalytic activity was measured in the supernatants. The used 50 mM buffer systems were: citrate-buffer (p*K*_1_ = 3.13, p*K*_2_ = 4.76) in the pH-range from 2.5 to 5.5, carbonate-buffer (p*K* = 6.35) between pH 5.3 and 7.3, phosphate-buffer (p*K* = 7.2) between pH 6.2 and 8.2, Tris-buffer (p*K* = 8.06) in the range from pH 7.1 to 9.0 and glycine-buffer (p*K* = 9.78) between pH 8.8 and 10.7. All samples were measured in triplicates.

#### 2.2.4 Stability of HRP in the presence of concentrated intermediate bulk components

To identify the intermediate bulk components which might cause product loss, a stock solution of HRP was prepared in deionized water (50 U/ml) and mixed with aqueous stock solutions of the different components to be tested (Table 2) to a final concentration of 1 U/ml. The concentration of the stock solution was limited by the solubility of the respective substance and all solutions were set to pH 6.5 with either HCl [10 M] or KOH [10 M], whereby the risk of small influences caused by the added ions was taken into account.

#### 2.2.5 Detailed analysis of deactivating bulk media components

**Supplementary Fig. S1:**
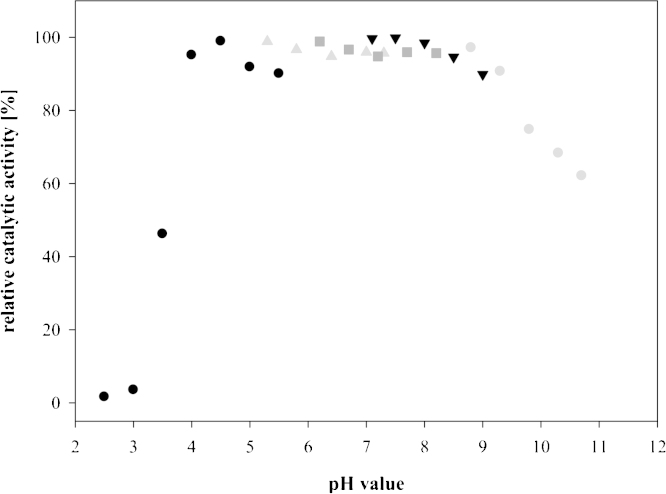
pH stability of HRP. ●, citrate-buffer; ▴, carbonate-buffer; ■, phosphate-buffer; ▾, TRIS-buffer; ●, glycine-buffer.

To minimize any possible deactivating effects of added acid or base, and thus additional ions, the pH value was only set to values between pH 4.0 and 9.0 for salt solutions, which exhibited a value outside this range after complete dissolution. Between pH 4.0 and 9.0 HRP had been determined to be stable and active, exhibiting more than 90% relative activity (Supplementary Fig. S1). In Table 3 the different salts, the concentrations and the pH values of the respective stock solutions as well as the analysis goals are given. For all stability measurements, HRP was added to the different stock solutions to a final concentration of 1 U/ml and incubated at 30 °C in a water bath for 30 min. The remaining enzymatic activity was compared to a standard (i.e. 1 U/ml HRP in 50 mM KH_2_PO_4_, pH 6.5). All samples were measured in triplicates.

### 2.3 Freezing study

#### 2.3.1 Packaging and freezing procedure

We investigated the freezing process in plastic bottles, which is a common procedure in scientific institutions and small enterprises due to flexibility and cost efficiency. High density polyethylene (HDPE) and glycol-modified polyethylene terephthalate (PETG) are widely used bottle materials, the latter having high impact strength at low temperatures and being well-studied with respect to chemical resistance [2,23]. In this study, cylindrical polycarbonate containers (Nalgene, VWR, 216-8207), which allow the removal of the frozen product by ice core drilling, were used. Polycarbonate was chosen due to its outstanding temperature stability. The thermal properties are identical to those of PETG (Supplementary Table S1), allowing direct comparison. The typical size in bottle freezing is between 0.5 and 2.0 L. In this study, a bottle size of 1 L was chosen. To prevent damage due to freezing-dependent volume increase, the filling level is usually set to only 80% of the total container capacity. Therefore, cylindrical containers with a diameter of 120 mm, a height of 145 mm and a total capacity of 1 L were used and filled with 800 ml intermediate bulk. For freezing we used a computer-controlled, liquid N_2_-operated lab-freezer with active cold gas circulation. Up to four containers were frozen simultaneously at a process temperature of −30 °C.

#### 2.3.2 Concentration of components in the frozen intermediate bulk

**Fig. 1:**
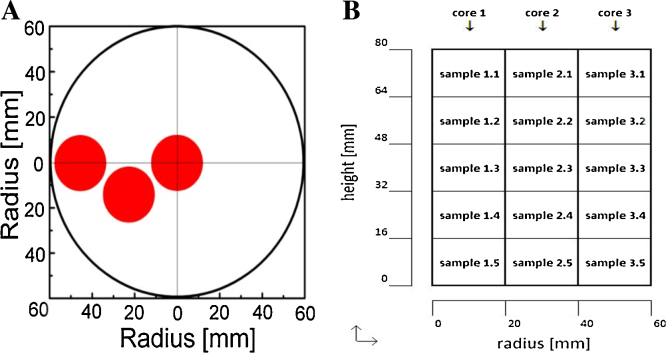
Sample preparation for the determination of concentration distribution in the frozen intermediate bulk. (A) Schematic top view of the freezing container showing the radial positions of ice core extraction; (B) vertical division of ice core samples.

After freezing, containers with frozen intermediate bulk were taken from the freezer and stored at −30 °C. To analyse local concentrations of intermediate bulk components, the containers were pre-tempered at −10 °C for 1 h to prevent crack formation during sampling. Subsequently, samples were taken with a 22 mm ice core drill. Three ice cores where extracted from every freezing container at different radial positions (Fig. 1A). The first core was taken adjacent to the container wall, the second at the half radial distance (hrd) with a tangential offset of approx. 25 mm and the third at the centre of the freezing container. After extraction, each ice core was vertically divided into 5 cylindrical blocks of equal length, resulting in 15 individual samples with a volume of approx. 3.2 cm^3^ (Fig. 1B). Samples were transferred into plastic tubes and defrosted at a defined temperature of 20 °C and radial shaking at 50 rpm. Subsequently, ionic concentrations of calcium, magnesium, potassium, phosphate (as phosphor) and sulphate (as sulphur) were measured by inducted coupled plasma optical emission spectroscopy (ICP-OES). Chloride concentration was measured by ion chromatography and Tris by quantitative HPLC. HRP concentration was analysed photometrically at a wavelength of 403 nm, which is the absorbance maximum of the heme-group, because protein concentration was below the detection limit of the Bradford assay. Enzymatic activity was measured as specified above.

#### 2.3.3 pH stability of the buffer system

To monitor pH shifts in the intermediate bulk during freezing, a preliminary examination was done using a simplified medium. All substances without significant contribution to the pH were omitted leaving only phosphate and Tris/HCl. To show the dependency between pH-stability and buffer concentration, 3 different concentrations of Tris/HCl, namely 0.1 M, 0.5 M and 1.0 M, were analysed. We prepared the respective Tris solutions, before phosphoric acid was added to obtain a phosphate concentration of 1.57 × 10^–2^ mol/l (see also Table 1) and then pH was adjusted to 7.0 by addition of HCl. These solutions were frozen as described above with the exception of a lower freezing temperature of −25 °C which was chosen for a more gentle treatment of the measuring system. The pH was monitored during the whole freezing process using a low temperature pH-electrode (InLab Cool, Mettler Toledo) with an added thermocouple for correction of temperature dependent characteristics of the measuring system. The pH electrode was placed in the centre of the freezing container. All measurements were done in duplicates.

#### 2.3.4 Spatiotemporal pH distribution in the intermediate bulk during freezing

**Fig. 2:**
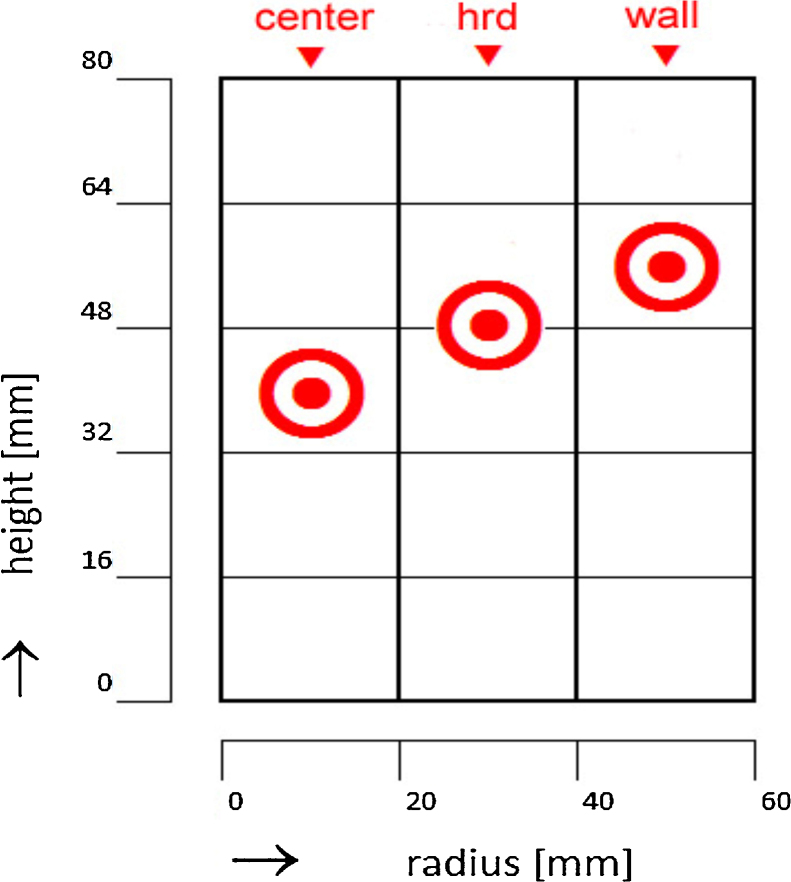
Vertical and radial diaphragm positions of the pH electrode in relation to the corresponding sample positions for the analysis of bulk component concentrations.

We monitored the spatiotemporal pH distribution in the intermediate bulk during freezing at −30 °C at three different positions in the freezing container (Fig. 2): in the centre of the freezing container at 50% filling height, at the hrd at 60% filling height, and near the container wall at 70% filling height. All measurements were done in triplicates. To prevent unwanted thermal influences, the pH electrode and electrode wire were double-insulated.

## 3 Results and discussion

Scientific literature proposes several factors which potentially damage proteins in aqueous solutions during freezing. One of these factors is “cold denaturation” which describes a direct impact of low temperatures on the protein [24,25]. Lowering the temperature means a decrease of intrinsic energy which in turn lowers hydrophobic interactions leading to structural protein alterations. However, this effect is very weak and was found to be completely reversible [24,25]. Another factor which can cause product loss is known as “concentration effects” [24–27]. Concentration effects are caused by different solidification characteristics of solvent and solutes. In aqueous buffers, water freezes first, while salts are concentrated to their individual solubility limits. These spatiotemporal concentration shifts cause changes in electrostatic and hydrophobic forces which irreversibly damage protein structures. Another parameter that is linked to concentration shifts, and therefore must be considered, is the pH value which directly influences protein stability.

In the present study, we analysed the effect of concentration and pH shifts in an intermediate bulk containing a biopharmaceutically relevant protein during freezing. The solvent in the intermediate bulk was water and the solutes were the product HRP, Tris/HCl buffer salts as well as residuals from the cultivation broth (Table 1).

### 3.1 Characterization of HRP in aqueous solution

#### 3.1.1 Stability at 30 °C and pH stability

To exclude effects on product stability caused by the activity assay itself, stability of HRP at 30 °C was analysed. HRP was completely stable for more than 4 h. Only at the end of incubation a slight reduction of the relative catalytic activity to around 85% was observed (Supplementary Table S2). Thus, HRP stability was high enough to choose an incubation time of 30 min for all subsequent aqueous stability studies at 30 °C without running the risk of deactivating HRP only due to the incubation procedure itself. We also tested the stability of HRP between pH 2.5 and 10.7. Buffer concentrations were chosen with only 0.05 M to minimize the risk of a direct influence of buffer ions on protein stability. As shown in Supplementary Fig. S1, HRP showed high stability between pH 4.0 and 9.0. Only at lower and higher pH values, a significant reduction of enzymatic activity was observed.

#### 3.1.2 Stability of HRP in the presence of concentrated intermediate bulk components

Not all components in the intermediate bulk may harm the product during freezing. Hence, we identified bulk components which were potentially harmful for HRP in a fast screening approach. Therefore, HRP stability was tested at the maximum soluble concentrations of all bulk components. Tris and salts containing the ions potassium, calcium, chloride, sulphate and phosphate were dissolved in water to their solubility limit (Table 2). pH was set to 6.5, where HRP was found to be stable (Supplementary Fig. S1), and HRP was incubated in these salt solutions at 30 °C for 30 min before remaining catalytic activity was measured. HRP did not show any reduction in catalytic activity in the presence of either MgSO_4_·7H_2_O [2.5 M] or K_2_SO_4_ [1.67 M]. Hence, we concluded that HRP was stable in the presence of high concentrations of the intermediate bulk components potassium (up to 3.33 M), magnesium (up to 2.5 M) and sulphate (up to 2.5 M). However, when incubated in the presence of Tris [3.3 M] only around 8% relative catalytic activity were left, and in the presence of either CaCl_2_ [5.0 M] or KH_2_PO_4_ [1.6 M] no more catalytic activity was determined. This means, that Tris as well as calcium, chloride and phosphate could potentially cause HRP loss when they are concentrated during freezing. Thus, we focused on these bulk components in the subsequent freezing study.

### 3.2 Freezing study

#### 3.2.1 Concentration of components in the frozen intermediate bulk

**Fig. 3:**
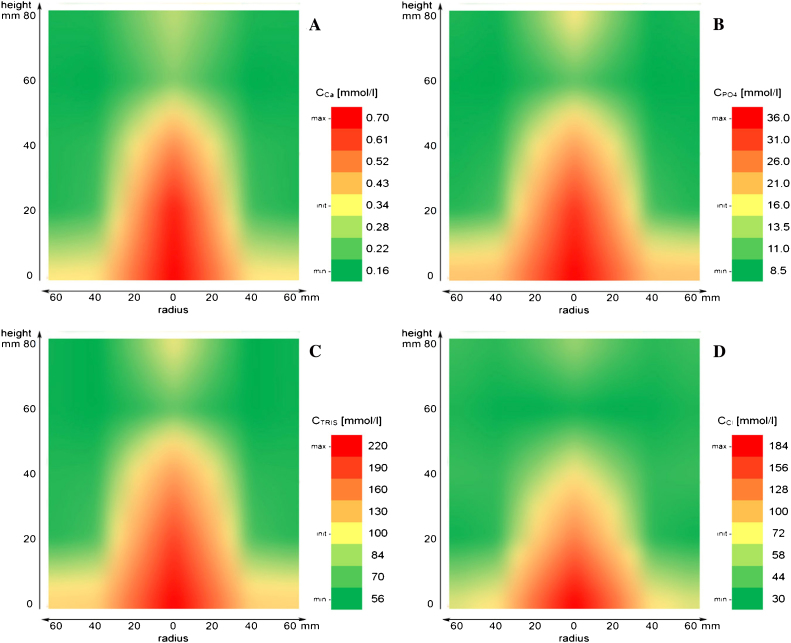
Concentration distribution shown for the cross-sections of the cylindrical freezing container of (A) calcium; (B) phosphate; (C) Tris; (D) chloride. Distribution functions were smoothed on the basis of 25 single values each. (For interpretation of the references to colour in text near the figure citation, the reader is referred to the web version of this article.)

We analysed the effect of freezing in 1 L bottles at −30 °C on the concentration of the bulk components Tris, calcium, chloride and phosphate and potential deactivating effects on the product HRP. Fig. 3 shows the concentration distribution of these components for the cross-section of the frozen medium cylinders. All diagrams are scaled between the minimum (green) and maximum concentration (red) locally found in the frozen bulk and centred to the initial concentration (yellow) before freezing. Despite small deviations, all components showed the same distribution profile. Horizontally, the lowest concentration was found near the walls while the maximum was found in the centre of the freezing container. Vertically, however, maximum concentrations were not found in the centre but shifted towards the bottom. This basic pattern can be explained by an overlay of different redistribution processes. In general, freezing is based on thermal conduction driven by heat transfer through the container walls causing a transient temperature gradient. This results in dendritic ice growth from the container walls to the centre. Progressing solidification of water leads to an increasing concentration of all bulk components that ideally should cause a symmetrical concentration increase between the walls and the centre. The downshift of the concentration maximum towards the bottom, which we observed here, may be explained by a simultaneous detachment of small branches from growing ice dendrites. Due to their lower density, detached branches of aqueous ice move upwards, displacing the concentrated bulk medium towards the bottom [28]. Both processes overlay and lead to the typical concentration redistribution scheme found here (Fig. 3). Only slight differences were found between the distribution profiles for calcium, phosphate and Tris. Chloride, however, showed a stronger accumulation in the lower part of the container.

For all components, the lowest and highest local concentrations were compared to the respective initial concentration in the intermediate bulk. After freezing, concentrations of only 40%, but also as high as 250% of the initial concentrations were measured (Supplementary Table S3). The highest concentration shift was found for chloride, while calcium showed the smallest difference between minimum and maximum concentrations, followed by Tris and phosphate. These findings nicely demonstrate that significant concentration effects happen during freezing. These concentration effects might affect product stability, even if no component in the intermediate bulk is harmful at its initial concentration. Moreover, it is shown that every bulk component undergoes a specific concentration shift during freezing. This behaviour can be explained by different entrapment rates of dissolved bulk components in the growing ice structure which is determined by specific solubility and solidification behaviour. Consequently, different concentration ratios are found at each position in the freezing container.

**Fig. 4:**
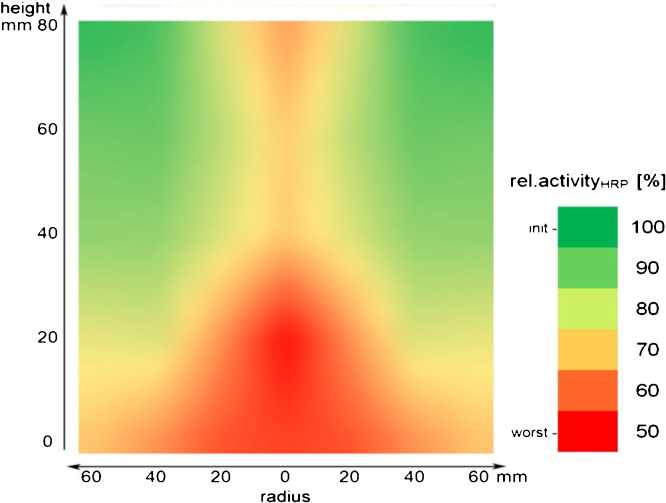
Distribution of residual HRP activity in the cross-section of the cylindrical freezing container after freezing. Distribution function was smoothed on the basis of 25 single values each.

To analyse if the product meets deactivating conditions during freezing and where these conditions might be more pronounced, we measured remaining HRP activity after freezing in the cross-section of the frozen intermediate bulk (Fig. 4). While concentration maxima of bulk components were found in the central bottom region (Fig. 3), maximum product loss was shifted upwards to the half height between the bottom and the centre (Fig. 4). This already indicated that product loss was not only caused by high concentrations of bulk components, but that a synergistic effect must have caused enzyme inactivation. Thus, we also analysed local changes of the pH value during freezing.

#### 3.2.2 pH stability of the buffer system and the intermediate bulk during freezing

**Supplementary Fig. S2:**
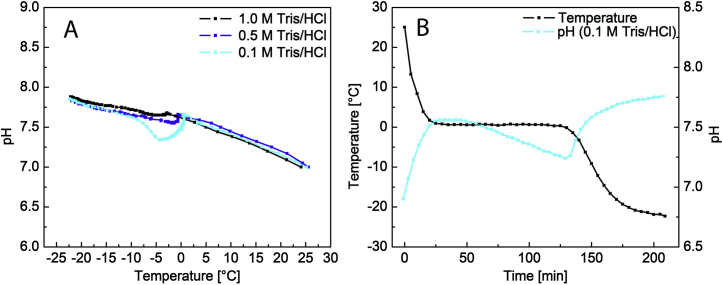
pH shifts during freezing of pure Tris/HCl buffer measured at three different concentrations (initial pH value was pH 7.0).

**Supplementary Fig. S3:**
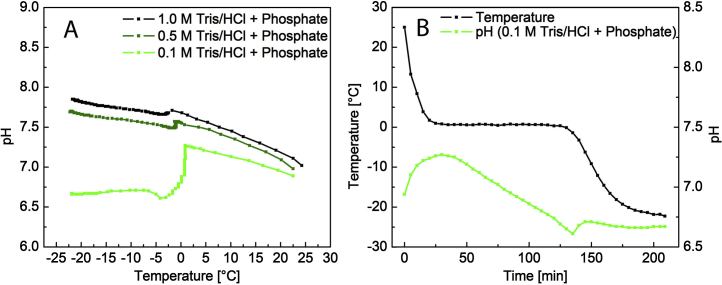
pH shifts during freezing of Tris/HCl buffer plus 15.7 mM phosphate measured at three different TRIS concentrations (initial pH value was pH 7.0).

Tris is a common buffer for enzyme processing since it does not inhibit enzymes at typical concentrations, but rather has stabilizing effects [29]. Thus, Tris/HCl was chosen for pH stabilization of the intermediate bulk. However, previous studies have reported significant pH shifts during freezing of Tris which is why we analysed potential pH shifts of differently concentrated Tris/HCl buffers during freezing. Independent of the molar Tris concentration we monitored a constant pH increase at a rate of approx. −0.026 pH units per degree Celsius during the whole freezing process (Supplementary Fig. S2). This might be explained by the strong temperature dependency of the dissociation constant for Tris which is given with −0.028 to −0.03 pH units per K [30]. Below 0 °C the pH value decreased in a concentration dependent manner before it stabilized (e.g. at −5 °C for the 0.1 molar buffer) and increased again with progressing solidification. In literature, such freezing-related pH changes are described as pH shifts [31–33]. Most authors ascribe this phenomenon to a precipitation of buffer salts during freezing and a subsequent reduction of buffering capacity. This common explanation considers the freezing process as a local solidification event, in which water starts to solidify below the freezing point until solutes reach their solubility limit which is why buffer concentration increases before precipitation. As a consequence, stabilization of the pH would be expected at the beginning of the freezing process and no pH decrease should happen above the eutectic temperature, which is −5.3 °C for Tris [33]. However, pH in the 0.1 M Tris/HCl buffer already decreased at a higher temperature than −5.3 °C (Supplementary Fig. S2). To explain this phenomenon, we have to consider the freezing process as a spatially progressing event rather than a local solidification event. Below 0 °C, formation of ice starts at the walls of the freezing container before it slowly grows towards the centre. In fact, it takes about 2 h until ice formation starts there. During this whole time, pH constantly decreases in the fluid bulk medium. This clearly indicates that the pH shift is caused by a constant alteration of the ion concentration ratios in the residual fluid during freezing: due to better solubility, chloride is concentrated to a larger extent than Tris causing the observed pH shift. For 0.1 M Tris/HCl, pH stabilized just at the moment when the temperature drop indicated local solidification. After stabilization, we expected another pH drop caused by an unequal precipitation of buffer components, before pH was supposed to increase again. However, for 0.1 M Tris/HCl the second pH drop was not detected (Supplementary Fig. S2), because it was overlaid by the strong temperature-dependent pH increase of Tris. However, upon phosphate addition the expected freezing behaviour was clearly seen (Supplementary Fig. S3). Although the final pH value at the end of the solidification process was the same for 0.1 M, 0.5 M and 1.0 M Tris/HCl, we experienced a more pronounced pH shift with the 0.1 M Tris/HCl buffer system. This underlines the importance of the ionic strength of the buffer used in freezing processes.

**Supplementary Fig. S4:**
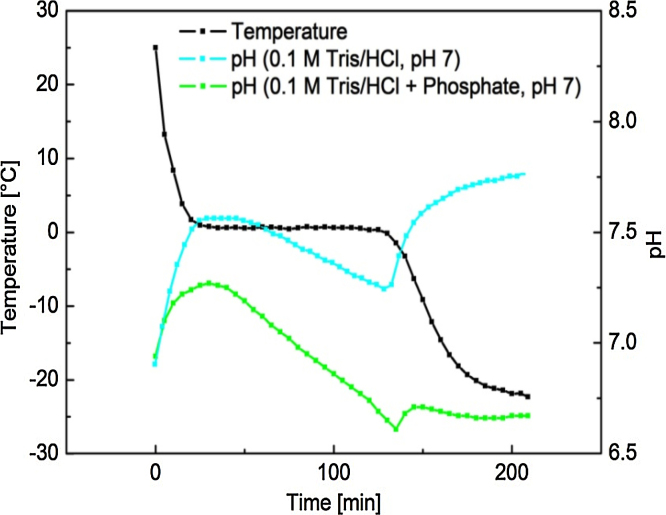
pH-shift and temperature in the centre of the freezing container during freezing of both 1 L 0.1 M Tris/HCl and 1 L 0.1 M Tris/HCl plus 15.7 mM phosphate at −30 ̊C plotted as a function of process time.

However, after diafiltration, the intermediate bulk does not only contain Tris but also a serious amount of residual phosphate (see also Table 1). To simulate the higher complexity of the intermediate bulk, we added phosphate to a final concentration of 15.7 mM to the different Tris/HCl buffers and repeated the freezing experiment (Supplementary Fig. S3). For 0.5 M and 1.0 M Tris/HCl plus phosphate, the pH alteration during freezing was almost similar to that of the Tris/HCl buffer. However, for the 0.1 M Tris/HCl buffer system the pH shift changed significantly upon phosphate addition (compare Supplementary Figs. S2 and S3). Not only the rate of pH increase was significantly lower, but also the pH-shift below 0 °C was much more pronounced. As mentioned above, we found an intermediate pH stabilization followed by a second pH decrease, but no subsequent pH increase happened at the end of the solidification process. This different freezing behaviour can be explained by the dissociation equilibrium for Tris/HCl and phosphoric acid. Below pH 7.0 the low buffer capacity of 0.1 M Tris/HCl was increasingly depleted, while phosphate with a p*K*_2_ of 7.21 showed equilibrium reaction. So, with decreasing pH the negative temperature shift of the p*K* of the Tris/HCl buffer lost influence while the curve slope got increasingly dominated by the p*K*_2_ of phosphoric acid, which shows no temperature dependency [34]. Consequently, compared to the 0.1 M Tris/HCl buffer system, 0.1 M Tris/HCl plus phosphate showed poor pH stability during freezing. To illustrate this significant difference better, the pH shifts for 0.1 M Tris/HCl and 0.1 M Tris/HCl plus 15.7 mM phosphate in the centre of the freezing container over time are compared in Supplementary Fig. S4.

#### 3.2.3 Spatiotemporal pH distribution in the intermediate bulk during freezing

**Fig. 5:**
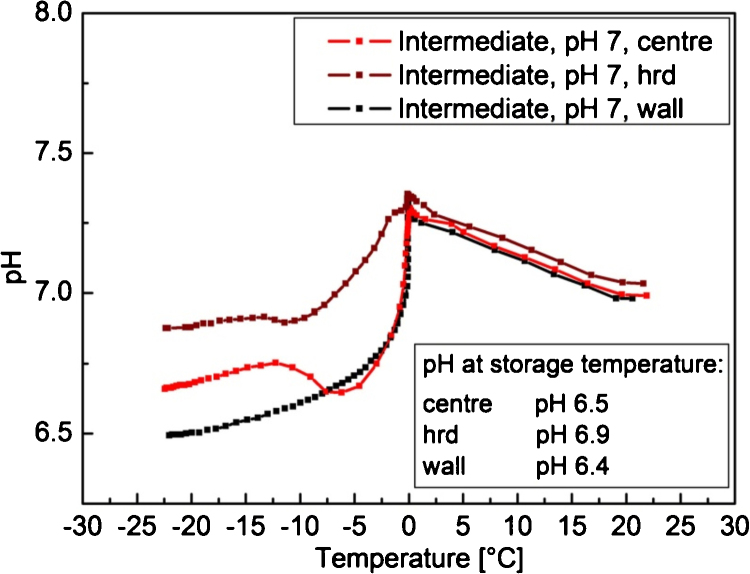
Spatiotemporal measurements of pH shifts during intermediate bulk freezing. pH was recorded at three different positions in the freezing container: in the centre of the freezing container (centre), at the upper container wall (wall) and in the horizontal and vertical middle of these positions (hrd = half radial distance).

Monitoring pH during freezing of either 0.1 M Tris/HCl or a simplified bulk medium showed a pH-shift in the centre of the freezing container which was related to a significant concentration shift of chloride. In the frozen bulk we found different concentration ratios in the freezing container which probably resulted from specific entrapment rates for dissolved bulk components during ice formation (Fig. 3). Thus, we assumed also different pH values at different positions in the freezing container. To prove this presumption the pH value was measured at three different positions between the symmetrical centre and the upper wall of the freezing container (centre, hrd, wall; Fig. 2) during freezing. The respective pH shifts at the different positions during freezing are shown in Fig. 5. During cooling to the freezing point, identical curve slopes were found at all measuring positions. However, with beginning of solidification we observed significant differences in the strength and the kinetics of pH alteration (Fig. 5). In the centre we found pH shifts identical to 0.1 M Tris/HCl plus phosphate. At half radial distance (hrd) between the centre and the wall we observed a similar pH shift only not as pronounced, which is why the final pH at this location was significantly higher. However, at the upper container wall a different curve shape was observed. Although pH also decreased below 0 °C, a subsequent stabilization and another pH drop did not happen. This deviation can be explained by different process kinetics at the wall. As discussed before, freezing has to be considered as a two-step process. In the first step, ice formation in the outer area of the container shifts the pH of the fluid medium. The second step is characterized by local ice formation, which causes a temporary pH stabilization that is followed by another pH shift due to precipitation of buffer salts. At the container wall, however, the bulk medium is in direct contact with the surrounding cold gas and local ice formation starts immediately. Hence, the first step of the two-step freezing process does not happen but only a single pH shift which is more pronounced due to the reduced buffering capacity. Summarizing, it is obvious that the pH value undergoes a complex redistribution process during freezing resulting in distinct local minima and maxima.

#### 3.2.4 Synergistic effects on product stability

**Fig. 6:**
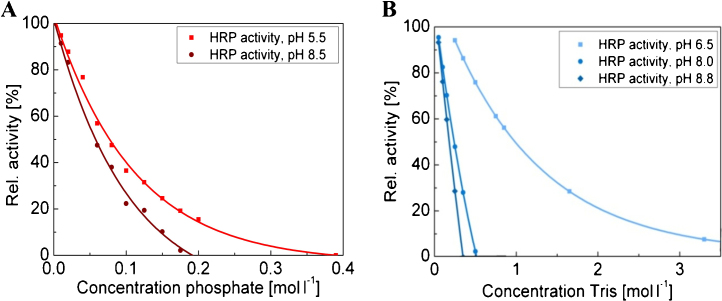
HRP activity measured at different pH values in the presence of different concentrations of either (A) phosphate, or (B) Tris.

To evaluate synergistic effects of ion concentrations and pH on product stability, we analysed HRP inactivation in different buffers at different pH values. At first, the intermediate bulk components with the strongest influence on the product were identified by comparative measurements of HRP activity in the presence of rising concentrations of Tris, CaCl_2_ and KH_2_PO_4_ at pH 6.5 (data not shown). Tris and phosphate were identified as the primary cause for inactivation. Subsequently, HRP inactivation was investigated in the presence of these two components in a concentration range which was found in the frozen intermediate bulk at different pH values, depicting the pH values found in the buffer freezing experiments. As shown in Fig. 6, there were in fact synergistic effects of ion concentration and pH causing product loss. Apparently, HRP was more stable against higher ion concentrations at acidic than at alkaline pH values. This was nicely demonstrated by the inactivation experiments in Tris, as Tris caused no HRP activation at pH 6.5 even for the maximum concentration that was found in the frozen bulk, but approx. 45% product loss at pH 8.0 and even 60% product loss at pH 8.8. From this point of view, Tris does not seem to be a suitable storage buffer for HRP, even if it is an excellent storage buffer for other proteins and is thus often used [29].

The synergistic effect of ion concentration and pH was not only evident in simple buffer systems (Fig. 6), but also in the frozen intermediate bulk as the concentration distribution of protein damaging ions and the distribution of product loss showed different patterns. With respect to local ion concentrations in the container, product loss at the half radial distance (hrd) was much more pronounced compared to the centre of the freezing container or the wall position. This can be ascribed to pH effects, as pH was acidic at the wall and centre position, but significantly higher at hrd.

## 4 Conclusions

The main goal of the present study was to obtain basic understanding of protein degradation during an intermediate bulk freezing process. Summarizing, this study shows that freezing of a buffered intermediate bulk describes a very complex process where product loss is caused by a synergistic effect of ion redistribution and pH changes. Media components and buffer salts experience great concentration during freezing up to levels which harm the product, even if their concentrations in the unfrozen bulk are harmless. Due to concentration effects, also pH in the freezing bulk changes. Even if pH minima and maxima per se do not harm the product, the changed pH might intensify the damaging effect of the concentrated ions. Thus, product loss during freezing is caused by a complex synergistic effect of local ion concentrations and pH values. Unfortunately, both parameters are caused by a cascade of interacting physical processes and do not show the same redistribution pattern, which makes it impossible to predict the total product loss during freezing only by reducing the freezing temperature. The freezing of complex bulk solutions can only be optimized by scientific characterization and simulation of the freezing process. Only if the local redistribution of both ion concentrations and pH values are known, product loss may be calculated and minimized by optimizing the freezing process. Concentration effects are specific for the composition of the medium and the buffer and have to be determined on a case-to-case basis.

## Conflict of interest

The authors declare that they have no conflict of interest.

## Figures and Tables

**Table 1:** Composition of the intermediate bulk after diafiltration.

Intermediate bulk component		Concentration
Product	HRP	50.0 mg/l
Residuals from cultivation broth	Magnesium	1.62 × 10^–3^ mol/l
	Potassium	1.04 × 10^–2^ mol/l
	Calcium	3.40 × 10^–4^ mol/l
	Sulphate	7.18 × 10^−3^ mol/l
	Phosphate	1.57 × 10^–2^ mol/l

Buffer (0.1 M Tris/HCl; pH 7.0)	Tris(hydroxymethyl)aminomethane	1.00 × 10^–1^ mol/l
	Chloride	7.25 × 10^–2^ mol/l

**Table 2:** Concentrated stock solutions of bulk components for stability tests of HRP.

Bulk component	Concentration [M]
K_2_SO_4_	1.67
MgSO_4_·7H_2_O	2.5
CaCl_2_	5.0
Tris	3.3
(NH_4_)_2_SO_4_	3.3
KH_2_PO_4_	1.6

**Table 3:** Concentrated salt stock solutions for stability tests of HRP. pH values were set between pH 4.0 and 9.0.

Salt	Concentration [M]	pH	Check denaturing effect of
Tris	3.3	6.5^a^ 8.0^a^ 8.8^a^	Tris pH value
CaCl_2_	4.0	4.7	Ca^2+^ or Cl^–^ ions
KH_2_PO_4_	3.6	5.5^a^ 8.8^a^	PO_4_^3−^ ions pH value

a pH was set with KOH [10 M] or HCl [10 M].
