# Supplementary material for: Examining the freezing process of an intermediate bulk containing an industrially relevant protein
Source: Enzyme Microb Technol. 2015 Apr;71:13–9. doi: 10.1016/j.enzmictec.2015.01.003 (PMC4370381; doi:10.1016/j.enzmictec.2015.01.003)
Supplement: Supplementary file 1 [file mmc5.pdf]

Supplementary Data

Supplementary Table 1. Thermal properties of polyethylene terephthalate (PETG) and polycarbonate (PC).

**Property**	**PETG**	**PC**
spec. heat capacity	1.1 J/gK	0.96 - 1.17 J/gK
thermal conductivity	0.2 W/mK	0.2 W/mK
temperature resistance	no data available	up to -135 °C

Supplementary Table 2. Stability of HRP [1 U/ml] in KH_2PO_4 (50 mM, pH 6.5) at 30 °C.

**time [h]**	**catal. activity [U/mL]**	**rel. activity [%]**
0	0.967 ± 0.090	100
2	1.007 ± 0.021	104
4	1.017 ± 0.015	105
6	0.822 ± 0.013	85

Supplementary Table 3. Local maximum and minimum concentrations of bulk components in the frozen intermediate bulk compared to initial concentrations in the unfrozen state.

**bulk component**	**initial conc.** [M]	**local min. conc.** [M]	**decrease** [%]	**local max. conc.** [M]	**increase** [%]
Ca^{2+}	$3.4{\cdot}10^{-4}$	$1.8{\cdot}10^{-4}$	52	$6.5{\cdot}10^{-4}$	191
PO_4^{3-}	$1.57{\cdot}10^{-2}$	$8.52{\cdot}10^{-3}$	54	$3.56{\cdot}10^{-2}$	226
Cl^{-}	$7.25{\cdot}10^{-2}$	$3.11{\cdot}10^{-2}$	43	$1.83{\cdot}10^{-1}$	252
TRIS	$1.00{\cdot}10^{-1}$	$5.9{\cdot}10^{-2}$	59	$2.16{\cdot}10^{-1}$	216
